# The “child size medicines” concept: policy provisions in Uganda

**DOI:** 10.1186/s40545-015-0025-7

**Published:** 2015-01-31

**Authors:** Xavier Nsabagasani, Jasper Ogwal-Okeng, Anthony Mbonye, Freddie Ssengooba, Rebecca Nantanda, Herbert Muyinda, Ebba Holme Hansen

**Affiliations:** Child Health and Development Center, College of Health Sciences, Makerere University, Kampala, Uganda; Department of Pharmacology and Therapeutics, Gulu University, Gulu, Uganda; Department of Community Health, Ministry of Health Uganda, Kampala, Uganda; School of Public Health, College of Health Sciences, Makerere University, Kampala, Uganda; Department of Pharmacy, Section for Social and Clinical Pharmacy, University of Copenhagen, Universitetsparken, Copenhagen, Denmark

**Keywords:** Essential medicines, ‘Child size medicines’, Guidelines, Policy, Uganda

## Abstract

**Background:**

In 2007, the World Health Organization (WHO) launched the ‘make medicines child size’ (MMCS) campaign by urging countries to prioritize procurement of medicines with appropriate strengths for children’s age and weight and, in child-friendly formulations of rectal and flexible oral solid formulations. This study examined policy provisions for MMCS recommendations in Uganda.

**Methods:**

This was an in-depth case study of the Ugandan health policy documents to assess provisions for MMCS recommendations in respect to oral and rectal medicine formulations for malaria, pneumonia and diarrhea, the major causes of morbidity and mortality among children in Uganda- diseases that were also emphasized in the MMCS campaign. Asthma and epilepsy were included as conditions that require long term care. Schistomiasis was included as a neglected tropical disease. Content analysis was used to assess evidence of policy provisions for the MMCS recommendations.

**Results:**

For most medicines for the selected diseases, appropriate strength for children’s age and weight was addressed especially in the EMHSLU 2012. However, policy documents neither referred to ‘child size medicines’ concept nor provided for flexible oral solid dosage formulations like dispersible tablets, pellets and granules- indicating limited adherence to MMCS recommendations. Some of the medicines recommended in the clinical guidelines as first line treatment for malaria and pneumonia among children were not evidence-based.

**Conclusion:**

The Ugandan health policy documents reflected limited adherence to the MMCS recommendations. This and failure to use evidence based medicines may result into treatment failure and or death. A revision of the current policies and guidelines to better reflect ‘child size’, child appropriate and evidence based medicines for children is recommended.

## Introduction

The need for appropriate medicines for children has attracted attention worldwide [1-5]. It is argued that appropriate medicine formulations should be the basis for drug therapy for children to ensure efficacy and safety [5,6]. Unsuitable formulations may lead to the child not taking the medicines, or receiving inappropriate doses leading to adverse reactions or ineffective treatment [7], needless to mention death. Many formulations used for children, especially tablets, are inappropriate for dosing, dispensing and administering [8]. For example, tablets for adults have traditionally been split and given to children, resulting in inaccurate doses in view of the children’s weight, age, physiological and cognitive conditions [9].

The World Health Organization in 2007, launched the ‘make medicines child size’ (MMCS) campaign to ensure that children receive the right medicine in the right dose. The MMCS initiative defined ‘child size medicines’ as those with: appropriate strengths and, child-friendly characteristics such as suppositories, solutions and flexible solid oral dosage formulations [10]. The United Nations (UN) member states were urged to make the procurement and supply of ‘child size medicines’ a priority and also ensure that there are corresponding legislative and regulatory measures for safe medicine use among children [11]. WHO also accentuated the importance of children’s medicines being based on the recent evidence of efficacy and safety for treating the specified children [10]. Flexible solid oral dosage forms are considered most suitable for children at the global level especially for developing countries [12]. The flexible solid oral dosage forms include tablets that are dispersible and can be used for preparation of oral liquids suitable for the younger age groups, powders, granules and pellets [10]. Evidence based medicine has been defined by Dickerson and others as a healthcare practice that is based on integrating knowledge gained from the best available research evidence, clinical expertise, patients' values and circumstances [13]. In 2011, WHO, with support from the United Nations Fund for Population Activities (UNFPA) and United Nations International Children’s Emergency Fund (UNICEF) proposed a list of lifesaving priority medicines for mothers and children to reduce maternal, new born and under five morbidity and mortality [14,15].

The role of policy provisions in increasing children’s access to appropriate medicines, as recommended by the MMCS campaign, cannot be underestimated. The need for country level reviews about the status of pharmaceutical policies, practices and the degree to which these policies are adhered to has been underscored [16]. It has been argued that evaluation of national medicine policies has potential for creation of evidence that other countries can use to formulate similar policies in their settings [17].

A study of provisions for priority medicines for mothers and children in the national essential medicines’ lists concluded that countries need to urgently amend their lists to provide all priority medicines as part of the efforts to improve maternal and child health [18]. Studies about the implementation of ‘child size medicine’ policies at country levels have been scarce. A survey by WHO in 14 African countries recommended an improvement of access to medicines for children [19]. In Tanzania, it was reported that caretakers experienced problems in administering tablets whereby the children either disliked the taste or vomited the medicines [20]. A study on provisions for safe medicines for children in Nigeria, on the other hand, found that there was a lack of paediatric focus in the essential medicines lists, a lack of access to up-to-date medicine information and weak national level policies [21].

There have been variations in the definition of policy. However, policy is commonly referred to as: formal authorization [22] norms, values and power [23] or ‘course of action that affects a set of institutions, organizations, services and funding arrangements [24]. This study collectively refers to policy statements, strategies, lists of essential medicines and clinical guidelines as ‘policy documents’.

In Uganda, the essential medicines management program has been in place since 1985 [25] and has been influential in determining the medicines that are procured by public health facilities. Over the years, this program has evolved and is the origin of policy provisions like the essential medicines lists and clinical guidelines. Prior to and after the launch of the MMCS campaign, no studies tailored to policy provisions for child size medicines in Uganda existed. Although the medicine policy reforms affecting essential medicines lists and clinical guidelines have been introduced, it is not clear to what extent they have addressed the MMCS recommendations. The objective of this study was to investigate the policy provisions for MMCS campaign by WHO in Uganda and make recommendations on how to address the policy gaps, if any.

## Methods

### Study setting

Uganda is a low income African country with population of 34.9 million according to the population and housing census conducted recently [26]. The under-five mortality rate is 90 per 1000 live births and the infant mortality rate is 54 per 1000 live births [27]. The National Medical Stores (NMS) is an autonomous government department that procures and distributes medicines to public facilities. Another key distributor of medicines is the Joint Medical Stores (JMS) - an umbrella organization for the Uganda Catholic and Uganda Protestant medical bureaus. However, donor funded programs for malaria, the Integrated Community Case Management of fever (ICCM), HIV/AIDS and tuberculosis, also procure and distribute medicines.

The health care system is organized under a hierarchy of health facilities, the lowest being Health Centre I (HC I) and highest being the National Referral Hospital. A Health Centre I (HC I) is a village facility with no defined physical structure where community out-reach services take place. HC I is managed by the Village Health Team (VHT) volunteers who do health promotions, distribute some medicines and mobilise communities for utilization of health services. A Health Centre II (HC II) is the physical health service structure closest to the community that provides basic curative services. A Health Centre III (HC III) is a little more comprehensive than HC II providing basic preventive and curative care services, handles referrals from the HC II, but also refers to HC IV. A Health Centre IV (HC IV) is a mini hospital with a simple theatre for minor surgeries covering a population equivalent to a constituency. In the Ugandan decentralized system, this is referred to as the Health Sub-District (HSD). Above this level are district hospitals, regional and national referral hospitals.

### Study design

The key methodological approach was an empirical one: a review of 10 selected Ministry of Health policy documents. This was an in-depth case study of policy documents in Uganda whereby archived documents were retrieved from the Ugandan Ministry of Health (MOH) and development partners. The documents were reviewed to empirically verify the policy provisions for ‘child size medicines’. Initially, it was realised that there was no single MOH official with an overview of the relevant policy documents that would address our research question. There was no single office (including the MOH resource centre) that had an inventory of all the documents in one place. Therefore, the relevant policy documents were identified using a rigorous process of consultation with Ministry of Health officials, institutions and officials from the relevant departments, donor community and other development partners. Institutions consulted included the Child Health Division and Pharmacy departments of the MOH, NMS, JMS, National Drugs Authority (NDA), Malaria Consortium (MC), WHO, UNICEF, Securing Ugandan’s rights to Essential medicines (SURE) and STRIDES for family health. Other sources included libraries, resource centres of workers, archives in the MOH resource centre (the library and information centre) and references of relevant published literature.

The internet was searched including the Uganda MOH website [28]. On the MOH website, there was a section labelled ‘policy documents & guidelines’ with a link to the ministry’s publications. Search terms included: child size medicines, paediatric formulations, child-friendly medicines, treatment guidelines, policy statements, strategic plans, clinical guidelines and essential medicines lists. Guidelines of child health programs such as Integrated Management of Childhood Illnesses (IMCI), Integrated Community Case Management (ICCM) and malaria treatment policy guidelines were also scrutinized.

The criteria for inclusion of documents for further analysis were whether they covered the aspects of medicines for tracer conditions considered in the study, medicines used to treat these conditions, their dosage formulations and whether they were developed by or in partnership with the government between the year 2007 and 2013. Overall, twenty four documents were retrieved because their titles, headings and contents were found relevant using the quality control criteria of authenticity, credibility, representativeness and meaning as discussed by Scott [29]. However, only 10 documents qualified for further review after checking whether they mentioned child-size medicines, whether medicines they mentioned were child appropriate with strengths adjusted to children’s age and weight.

### Data analysis

Content analysis was used to assess provisions for ‘child size medicines’ and child-friendly formulations. The documents were checked for information about ‘child size medicine’ concept, medicines strengths for children based on scientific evidence, flexible oral dosage forms, dispersible and effervescent tablets, solutions, syrups and suppositories. Also considered were provisions for medicines for malaria, pneumonia and diarrhoea being the major causes of childhood morbidity and mortality in Uganda and being the key target diseases for the MMCS campaign [30]. Asthma and epilepsy were included because they are among the most common chronic childhood conditions in Uganda requiring long term management. Schistomiasis was considered as a neglected tropical disease with high prevalence in more than half of the districts in Uganda yet prevalence of the disease among preschool children is at 39.3% according to a recent study [31]. In this paper, we refer to these diseases as ‘tracer conditions’. We only included oral, inhaled and rectal formulations that are commonly prescribed to babies and usually administered by caretakers. The study excluded injectables because they are for specific disease severity and are administered by more trained personnel at different levels of health care.

All these content considerations were first combined into an assessment matrix which was finally translated into Tables 1, 2 and 3 developed in consultation with paediatricians and dispensers from Mulago National Referral Hospital. We applied the elements in the matrix to the documents, the medicines for the tracer conditions and the medicines recommended as vital for the various levels of the public health facilities in Uganda as reflected in Tables 1, 2 and 3.

**Table 1:** **Documents identified and their provisions for elements of ‘child size medicines’**

**Document**	**Year**	**Has strengths appropriate for children**	**Includes evidence based medicine**
The second National Health Policy (NHP II 2012/19)	2012	-	-
The Health Sector Strategic and Investment Plan 2010/11-2014/15	2010	-	+^1^
Health Sector Ministerial Policy Statement-FY2010 and 2011	2010	-	-
National Drug Authority Uganda Strategic Plan 2011-2015	2011	-	+^2^
Integrated Community Case Management of Childhood Malaria, Pneumonia and Diarrhoea: Implementation Guidelines	2010	-	+
The Uganda Clinical Guidelines	2010	+	-
The Uganda Clinical Guidelines	2012	+	-
The IMCI guidelines: Management of a sick child aged 2 months up to 5 years	−^3^	-	-
The Essential Medicine and Health Supplies List (EMHSLU)	2012	+	+
User’s Mannual: Use of Rapid Diagnostic Tests (RDTs) for Malaria in fever case management in Uganda.	2012	+	+

^1^The Health Sector strategic plan refers to the distribution of amoxicillin in the community by the village health team members. Amoxicillin is an evidence based medicine.

^2^National Drug Authority is referring to medicines that have been phased out such as chloroquine and fancidar in relation to treatment of malaria.

^3^This is a treatment chart for IMCI which has not been revised since 2002.

**Table 2:** **Child size medicines provisions for tracer conditions in the 2012 Essential Medicines and Health Supplies List of Uganda**

**Disease**	**Name of the medicines**	**Has appropriate strengths for children**	**Child-friendly dosage form**	**Evidence based medicine**	**Recommended as first line in the 2012 UCG**
Pneumonia	Cotrimoxazole	+	-	-	+
	Amoxicillin	+	-	+	-
Malaria	Rectal artesunate	+	+	+	-
	Artemether Lumefantrine	+	-	+	+
	Quinine	-	-	+	+
Diarrhoea	ORS	+	+	+	+
	Zinc sulphate	+	+	+	+
Epilepsy	Phenytoin	+	-	+	+
	Carbamazepine syrup	+	+	+	+
Asthma	Prednisolone	+	-	+	+
	Salbutamol nebulizer solution	+	-	+	+
Schistomiasis	Praziquantel	-	-	+	+

**Table 3:** **The Vital medicines for the tracer conditions at different levels of health care**

**Medicine**	**Child friendly**	**Appropriate strengths for children**	**Health facility level where the medicine classified as Vital**	**Comments**
Amoxicillin	_	+	HCII and above	Dispersible amoxicillin is provided at HC1 by the development partners. Although HC1 is not included in the VEN classification, amoxicillin is distributed there
Cotrimoxazole	-	+	HC II and above	Cotrimoxazole which is not evidence based is the recommended first line for pneumonia in public facilities
Artesunate	-	+	HC I and HCII	Both an injectable and rectal
Artemether lumefantrine	-	+	HC II	Dispersible Artemether lumefantrine is supplied by malaria consortium and UNICEF at HCI
Zinc sulphate	+	+	HC I and HCII	Is provided at HC1 by the development partners
ORS	+	+	HC I and HCII	Is provided at HC1 by the development partners
Praziquantel	-	-	None	Praziquantel is mainly provided for adults in the community and schools through mass drug administration. Children below school going age are not included in the distribution
Prednisolone	-	+	None	
Salbutamol	+	+	HC IV	Asthma cases are also handled at the lower level health facilities
Carbamazepine tablets	-	+	HC IV	Epilepsy cases are also handled at the lower level health facilities
Carbamazepine syrup	+	+	HC IV	Same as above

### Ethical considerations

The study was approved by the Higher Degrees, Research and Ethics Committee of Makerere University College of Health Sciences (MakCHS) and the Uganda National Council of Science and Technology (Ref: SS 2703). The heads of the respective department in the MOH and development partners who gave verbal permission to access the documents.

## Results

### Historical overview of the reforms

The results indicate that since 2007, Uganda had revised the Clinical Guidelines (UCG) twice, in 2010 and in 2012. The guidelines for Integrated Community Case Management (ICCM) of childhood illnesses were introduced in 2010. In 2012, a new essential medicines and health supplies list of Uganda (EMHSLU) was introduced. The EMHSLU 2012 was differentiated from the Essential Medicines List 2007 by the introduction of essential health supplies component and the Vital Essential and Necessary (VEN) classification of medicines. The “Vital” (V) medicines are used to manage life-threatening diseases, “Essential” (E) medicines are effective in management of less severe, but nevertheless, widespread illnesses and “Necessary” (N) medicines are used to treat diseases with less impact on the population or items with a high cost for marginal therapeutic benefit. The overall purpose for introducing the VEN classification was to enable health facility and ministry of health procurement officials to prioritize medicines to procure. All these medicine reforms after 2007 were opportunities for addressing the requirements of the MMCS campaign.

Table 1 provides a list of the 10 documents that were reviewed. None of the documents made reference to the ‘child size medicine’ concept. However, elements of medicines strengths appropriate for children were included in the EMHSLU 2012. Flexible oral dosage forms such as dispersible tablets were missing in the EMHSLU 2012 for all the medicines for the tracer conditions except for zinc sulphate effervescent tablet for diarrhoea. The National Drug Authority strategic plan only highlighted some medicines that had been phased out from the list of the approved medicines. In that regard, we noted that a few of the medicines recommended in the UCG of 2010 and 2012 had been phased out by the National Drug Authority (NDA). The EMHSLU and UCG emerged as the only documents with much content about children’s medicines.

#### The essential medicines lists

The EMHSLU 2012 does not mention ‘child size medicines’ specifically. A further examination was done to establish whether the EMHSLU included aspects of child-friendly formulations such as dispersible tablets, effervescent tablets, inhaler solutions, syrups, suppositories and medicines strengths for children.

### Child-friendly medicines

The EMHSLU had limited provisions for child-friendly dosage formulations for medicines for each of the tracer conditions studied. According to Table 2, four out of the 12 medicines assessed were in child-friendly dosage formulations. These included rectal artesunate for malaria, ORS for diarrhoea, zinc sulphate effervescent tablet and carbamazepine syrup for epilepsy. None of the medicines for pneumonia, malaria and schistomiasis were in flexible oral solid dosage forms such as dispersible tablets. It should be noted that syrups (which are child-friendly in terms of administration) for malaria and pneumonia that were in the 2007 EML were excluded in the EMHSLU 2012. Rectal artesunate which uses a child-friendly channel of drug administration was provided for in the EMHSLU and was appropriate for its purpose of pre-referral emergency treatment. The medicines for asthma were included in the EMHSLU 2012 as inhalations which are child-friendly and the recommended method of administration of asthma medicines. However, it was noted that the spacers, that are supposed to be used in administering the inhalations to the children, were not included in the health supplies section of the EMHSLU 2012.

### Appropriate medicine strengths for children

Appropriate medicine strengths for children were present in both the Essential Medicines List (EML) 2007 and EMHSLU 2012. Table 2 shows that 10 of the 12 medicines (83%) assessed for the tracer conditions in the EMHSLU 2012 had appropriate strengths for children. The exceptions were quinine for malaria and praziquantel for schistomiasis.

### VEN classification

As already indicated, the EMHSLU 2012 introduced the concept of Vital, Essential and Necessary (VEN) Classification. The VEN classification was established to help facilities prioritize medicines to order, considering the often limited funding that they have been allocated. Table 3 shows the ‘child size medicine’ formulations that were classified as ‘vital’ according to the different healthcare facilities. Here it must be mentioned that amoxicillin and artemether-lumefantrine were also recommended for the ICCM program at HC I (village level facilities). All the child-friendly medicines including carbamazepine syrup, rectal artesunate, zinc sulphate dispersible tablets and oral rehydration salts (ORS), were classified as vital for the tracer conditions at all levels of healthcare.

#### The Uganda clinical guidelines

The guidelines recommended splitting of adults’ tablets for children. Furthermore, some of the medicines recommended for treatment of malaria and pneumonia among children were evidence based medicines.

### Recommendation of splitting of adult medicines to give to children

Two editions of the Uganda Clinical Guidelines (UCG) were reviewed (2010 and 2012) and in both documents, a specific section on IMCI was included. In terms of dosage the UCG 2010 still emphasized the need for breaking adult medicines according to age and weight:

“For children who are 12 years and less, the guidelines recommend that: the dose should be stated in terms of body weight; where weighing is not possible, doses should be approximated from adult doses: < 5 years ¼ of the adult dose, 5–8 years ½ of adult dose and 9–12 years ¾ of the adult dose. In this case, it is anticipated that Health workers will weigh the child and calculate the dose based on the weight. It also means that in case of administering tablets, they have to determine what portion of the tablet is to be given to the child” (UCG 2010: xxiv-xxv) [31].

### Evidence based medicines

Some of the medicines recommended in the clinical guidelines as first line treatment for malaria and pneumonia were not evidence-based and there were discrepancies across the different sections of the guidelines. For example, in the IMCI section of UCG out dated medicines such as a combination therapy of chloroquine and sulphadoxine pyrimethamine (CQ + SP) was recommended for malaria, while cotrimoxazole was recommended for pneumonia. On the other hand, in the general section of UCG 2010, the evidence based Artemether based combinations (ACTS) were recommended for malaria among children less than five years. In the IMCI section of the same guidelines, a combination of CQ and SP was recommended and yet by the time of this study, oral chloroquine and sulphadoxine pyrimethamine had been phased out as medicines for treating malaria. Both the 2010 and 2012 versions of the UCG recommended cotrimoxazole as first line treatment for pneumonia in spite of the ICCM guidelines recommending amoxicillin for the treatment of pneumonia. These discrepancies within the UCG and other policy documents such as the ICCM guidelines demonstrate lack of harmonization of the policy documents regarding evidenced based medicines.

## Discussion

The policy documents did not mention the ‘child size medicines’ concept anywhere, indicating a lack of adequate and specific attention to the MMCS recommendations. While the problem of medicine strengths according to age and weight of the child had been addressed in the EMHSLU and partly the UCG, there were no provisions for the WHO recommended flexible oral solid dosage forms (except for zinc sulphate effervescent tablet for treating diarrhoea) for easy administration of the medicines to children. Some of the medicines recommended in the clinical guidelines for malaria and pneumonia were not evidence based, demonstrating gaps in policy provisions for appropriate medicines for children in Uganda.

### The WHO recommendations on child-size medicines remain an unfinished business

There was no explicit reference to the ‘child size’ medicine concept in the Ugandan health policy documents. While there are 17 varieties of oral paediatric formulations which are ready-to-use at the global level [4] and whereas professional organizations, the pharmaceutical industry, and some governments from high income countries have emphasized ‘child size medicine,’ there is no such emphasis in low income settings like Uganda. Only three low income countries (Ghana, India and Tanzania) worldwide have been supported to pilot MMCS by Bill and Melinda Gates Foundation [12]. Hence, there are no experiences and guidelines for scale up of the ‘child size medicine concept’ and child appropriate formulations in other low income countries.

Flexible oral solid dosage forms have been recommended as the most appropriate for children since they allow accurate dosing and easy administration of medicines to children [7]. For example, all the lifesaving priority medicines in tablet forms are supposed to be in flexible oral solid dosage formulations such as dispersible tablets [14,15]. In the EMHSLU 2012, none of the medicines for malaria and pneumonia were presented in flexible solid oral dosage forms such as pellets, granules and dispersible tablets. This could lead to ineffectiveness of the treatment [32]. The omission of dispersible tablets for artemether- lumefantrine (for malaria) and amoxicillin (for pneumonia) in the EMHSLU is a critical gap since the two diseases are the main causes of morbidity and mortality in Uganda. Despite amoxicillin being an efficacious medicine for pneumonia which is the leading killer disease in children world-wide and in Uganda, and despite the recommendation that it should be provided as a scored dispersible tablet in order to optimize the benefits [15], this has not been the case in Uganda.

An analysis demonstrated that the medicine for diarrhoea specifically ORS (powder) and zinc (effervescent tablet) which were included in the medicines EMHSLU 2012 were not only evidence based but were also in flexible oral solid dosage forms. WHO and UNICEF have listed zinc sulphate effervescent tablets and ORS among the priority medicines for children with diarrhoea [14,15]. However, it has not been established whether these medicines are available in the health facilities in Uganda or not. Asthma medicines such as oral predinisolone are not in a flexible oral solid dosage forms and, depending on the age and weight, they may require the child to swallow several bitter tablets at a time.

### Medicine strengths for children

One of the important study findings was that except for praziquantel, a distinction has been made between medicines strengths for adults and those of children, especially in the EMHSLU 2012. Therefore, except in very exceptional cases, in future there will no longer be need to split adult tablets to determine the appropriate dose for children as has been widely professed in the literature. The remaining question is whether the medicines with strength for children are being procured and made available.

While the medicine strength suitable for children has been provided for, there is still a challenge of children swallowing formulations that are not child-friendly and flexible. This is because the current clinical guidelines still emphasize the practice of splitting adult medicines to treat children. This demonstrates a lack of harmonization between the guidelines and the essential medicines list- two documents that are supposed to complement each other.

Praziquantel which is used to treat schistomiasis has been presented in an adult strength only. This is despite of the fact that schistomiasis has been highlighted by WHO as one of the priority neglected tropical diseases, with an existent prerequisite to focus on children’s needs. However, despite the presence of tablets with strengths adjusted to children in the world market [6], the 600 mg tablet of praziquantel still remains the only dose available for treating schistomiasis for both adults and children in Uganda. In most of the sub Saharan Africa, schistomiasis is treated through Mass Drug Administration (MDA) programs to adult populations and children in schools. Although schistomiasis prevalence has been established in the age group below four years, little has been done to provide appropriate medicine for this age group [6]. In terms of medicines strengths, there are no provisions of praziquantel for the lower age categories. In Uganda, a low uptake of praziquantel among school children, due to side effects, unpalatable taste and the big tablet size has been reported [33,34].

### Evidence based medicines

Inconsistencies in the sections of the clinical guidelines, especially regarding recommendations of medicines that have already been phased out due to resistance were noted. These inconsistencies often leave health workers confused since for the case of Uganda, those who provide services at the lower levels of health care are inadequately trained and for that reason they largely depend on the guidelines to treat.

Cotrimoxazole was recommended as the first line treatment for pneumonia although studies have shown that it is no longer effective, especially in high HIV prevalence settings including Uganda [35,36]. Amoxicillin which is recommended by the WHO as the most effective antibiotic for pneumonia, is a second line treatment in Uganda and is only used as first line if there is a wheezing problem [37]. According to UNICEF, 1.56 million lives of children could be saved globally if amoxicillin was available [38]. Similarly, a combination of chloroquine and sulphadoxine pyrimethamine was recommended for treatment of malaria yet it is no longer effective. Therefore there is need for due diligence in terms of providing relevant and accurate guidelines, increasing access to efficacious and child-friendly medicines. This can be achieved when there are policy provisions for evidence based medicines.

#### Study strengths and limitations

##### Study strengths

The scope of this study was the period from 2007–2013. This provided an ample time of 6 years since the launch of WHO/UNICEF campaign for countries like Uganda to operationalize the concept at the national level. In the same period, some medicine reforms such as the revision of clinical guideline and essential medicines list 2012 took place and this was an opportunity to assess whether the reforms integrated the recommendations of the MMCS campaign. This was a case study on Ugandan policy documents. The study also discovered that document analysis could be a stand-alone qualitative method of research [39,40]. Case study designs have been recommended for an in-depth analysis of the policies and be replicable to other situations [41]. Policy documents are critical for understanding the policy direction and implementation. Since the launch of the MMCS campaign in 2007, very few case studies using policy document review as a method of analysis have been done. Using the approach, this study contributes considerably to the understanding of the extent of policy provisions for ‘child size’ medicine in a low income country like Uganda.

The importance of essential medicines policies on the quality of medicines in low-income countries has been underscored [42]. This reiterates our emphasis about the need to address the existing gaps in provisions of child-size and child appropriate medicines in the EMHSLU and the UCG. A study in Nigeria indicated that weak national policies negatively affected access to appropriate medicines for children [21]. The essential medicines list was one of the key documents from which the derived substantial information to verify provisions for MMCS recommendations. The concept of essential medicines lists has been accepted worldwide as a powerful tool to promoting health equity and its impact is remarkable, as the essential medicines have proved to be one of the most cost-effective elements in health care. Lists of essential medicines also guide the procurement and supply of medicines in the public sector, schemes that reimburse medicine costs, medicine donations and local medicine production, and, furthermore, are widely used as information and education tools by health professionals [43]. At the global level, including high income countries, the WHO’s campaign ‘*make medicines child size*’ has produced substantive accomplishments in building improved foundations to improve mechanisms that will enhance children’s access to critical medicines in resource-limited settings. However, there continues to be a challenge of a sustained, programmatic commitment especially in low income countries [44].

##### Limitations

From this review of documents, it was not possible to determine the rationale for the selection of the medicines in the essential medicines list and the extent of their availability and utilization at health facilities. To this end, studies focusing on stakeholder perspectives on the choice of children’s medicines, the extent to which such medicines are available at a health facility level and caretakers’ experiences about child size medicines are needed. Studies will also be needed to assess the availability of ‘child size medicines’ at the health facilities. More studies are needed to explore care givers’ experiences in administering some of the available child size medicines.

A major limitation for this study was lack of existing global standard criteria for assessing ‘child size medicines’. We therefore recommend that more detailed and standardized criteria should be developed for streamlining of the ‘child size medicines’ in different settings. This would help in future to ensure that the medicines provided in policy documents and operational guidelines are ‘child size’. Such an index could be based on the WHO preliminary requests [45] which at the moment do not provide sufficient guidance.

Injections were also left out which are also important and highly utilized by healthcare staff in the management of childhood illnesses. We therefore recommend additional studies focusing on injections for children to try and establish how child friendly and staff friendly they are.

#### Policy implications

Failure to integrate the MMCS recommendations into the essential medicines lists and clinical guidelines implies that many of the children are not accessing appropriate medicines and hence appropriate treatment. This might result in disease severity, needless to mention the persistent high mortality rate. Lack of child-friendly formulations such as flexible oral solid dosage forms and syrups affect the optimisation of treatment of children and for long term medication scenarios, this might lead to resentment that may in turn lead to further complications. Hence this study advocates for a medicine policy framework that addresses these problems specifically.

## Conclusion

The study has shown that the concept of the child size medicines is not explicitly reflected in the policy documents and this undermines its implementation; and hence efforts towards reducing under-five mortality. This might explain why the aspect of the flexible oral solid dosage forms is not taken care of especially regarding medicines for malaria and pneumonia, the main killer diseases in children in Uganda. Since medicine strengths for children have now been addressed, it is foreseen that splitting adult tablets to give to children will reduce with time, especially if the essential medicine list is followed during procurement of medicines and clinical guidelines are revised.

### Recommendations

There is an urgent need for the Ministry of Health, Uganda, WHO and UNICEF to review why the ‘child size medicine’ concept has not been fully implemented, with a view of providing further guidance. There is also a need to identify and integrate more flexible oral solid dosage forms for the vital medicines for children including antibiotics for pneumonia, medicines for malaria, diarrhoea and schistomiasis, into the essential medicines list. These formulations can be in the form of dispersible tablets, pellets and granules in the essential medicines list. There is also need to regularly update the essential medicines lists and the clinical guidelines with evidence based medicines and to further harmonise the diverse guidelines used by health workers especially the IMCI charts, to avoid misleading information.
